# What macromolecular crystallogenesis tells us – what is needed in the future

**DOI:** 10.1107/S2052252517006595

**Published:** 2017-05-24

**Authors:** Richard Giegé

**Affiliations:** a Architecture et Réactivité de l’ARN, UPR 9002, Université de Strasbourg and CNRS, F-67084 Strasbourg, France

**Keywords:** crystal engineering, crystallization predictors, crystallogenesis, crystallizability, crowding, determinant and antideterminant, evolution, packing, self-assembly rules, supramolecularity, surface patches, symmetry and asymmetry

## Abstract

Crystallogenesis is a longstanding topic that has transformed into a discipline that is mainly focused on the preparation of crystals for practising crystallo­graphers. Although the idiosyncratic features of proteins have to be taken into account, the crystallization of proteins is governed by the same physics as the crystallization of inorganic materials. At present, a diversified panel of crystallization methods adapted to proteins has been validated, and although only a few methods are in current practice, the success rate of crystallization has increased constantly, leading to the determination of ∼10^5^ X-ray structures. These structures reveal a huge repertoire of protein folds, but they only cover a restricted part of macromolecular diversity across the tree of life. In the future, crystals representative of missing structures or that will better document the structural dynamics and functional steps underlying biological processes need to be grown. For the pertinent choice of biologically relevant targets, computer-guided analysis of structural databases is needed. From another perspective, crystallization is a self-assembly process that can occur in the bulk of crowded fluids, with crystals being supramolecular assemblies. Life also uses self-assembly and supramolecular processes leading to transient, or less often stable, complexes. An integrated view of supramolecularity implies that proteins crystallizing either *in vitro* or *in vivo* or participating in cellular processes share common attributes, notably determinants and antideterminants that favour or disfavour their correct or incorrect associations. As a result, under *in vivo* conditions proteins show a balance between features that favour or disfavour association. If this balance is broken, disorders/diseases occur. Understanding crystallization under *in vivo* conditions is a challenge for the future. In this quest, the analysis of packing contacts and contacts within oligomers will be crucial in order to decipher the rules governing protein self-assembly and will guide the engineering of novel biomaterials. In a wider perspective, understanding such contacts will open the route towards supramolecular biology and generalized crystallogenesis.

## Abstract 2

Although the general rules governing protein crystal growth are known and a diversified panel of crystallization methods is available, producing focused protein crystals remains challenging. In the future, the supramolecular vision of crystallogenesis will break disciplinary boundaries and unify the field in a wider perspective beyond crystallization.

## 1. Crystallogenesis in the time of physiology and chemistry   

Protein^1^ crystallization dates back to the 19th century, when physiology and chemistry were the leading sciences. The field started with the crystallization of various haemoglobins and plant globulins, and culminated in the period 1925–1936 with the crystallization of urease, pepsin and a few other enzymes (reviewed by Giegé, 2013 ▸). At this time crystallization was used as a purification tool and allowed James B. Sumner and John H. Northrop to show that the catalytic power of enzymes resides in the protein itself and not in organic catalysts adsorbed on the protein surface. Sumner and Northrop were rewarded with the 1946 Nobel Prize in Chemistry, which was shared with Wendell M. Stanley, who isolated *Tobacco mosaic virus* (TMV) in a crystalline form and subsequently showed that it retains activity after solubilization. These seminal discoveries opened the era of modern biochemistry and molecular biology. Furthermore, the observations on TMV meant the death of vitalism, when it became clear that viruses act as chemical molecules.

**Figure 1:**
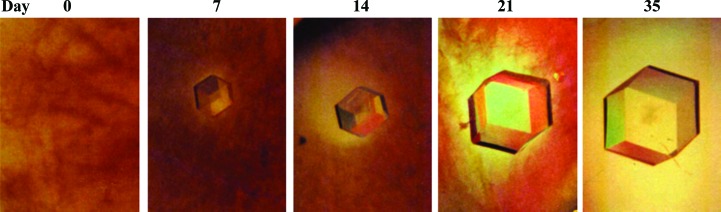
Ostwald ripening of a cubic TBSV crystal in a precipitate *in vitro* (courtesy of B. Lorber). The crystal grew in a 20 µl drop; after 35 d its volume reached the mm^3^ range. The clear halo around the crystal indicates that it grew at the expense of insoluble material. The images are at the same magnification.

Important influences on crystal science were the physicochemical studies on the solubility of proteins in salts by Franz Hofmeister and the work by Wilhelm Ostwald on the transformation/ripening of solid materials in solution, for which he was awarded the 1909 Nobel Prize in Chemistry. While the relevance to biology of Ostwald ripening remained elusive for years, its detection during protein crystallization came late and its physicochemical understanding is recent (Streets & Quake, 2010 ▸). A typical example of ripening was found when exploring the phase diagram of *Tomato bushy stunt virus* (TBSV; Lorber & Witz, 2008 ▸; Fig. 1 ▸).

A paradigm change occurred in 1934 with the first X-ray photographs of crystalline pepsin (Bernal & Crowfoot, 1934 ▸), with the focus of macromolecular crystallization moving rapidly from physiology and chemistry to biology and physics.

## 2. Crystallogenesis in the time of biology and physics   

### 2.1. A serious bottleneck when nascent molecular biology met X-ray crystallography   

In the early days of protein crystallography, when the methods for structure determination were first developed, the preparation of crystals appropriate for diffraction studies was not a major issue. The situation changed dramatically in the 1950s to 1960s, when the basic architectural elements of nucleic acids and proteins were discovered and when the nascent discipline of molecular biology aimed to understand the structure–function relationships in enzymology and the metabolic processes essential for life. This motivated structural biologists to attempt the targeted crystallization of enzymes, membrane proteins, tRNAs and supramolecular assemblies. However, given the multiparametric nature of the crystallization process and the difficulty in obtaining these compounds in the gram amounts required for the existing crystallization methods, it was rapidly realised that the growth of X-ray compatible crystals was a limiting factor. Thus, the current batch and dialysis methods were scaled down and vapour-diffusion and interface-diffusion methods were invented (Table 1 ▸). At the same time, new purification technologies were developed. Together, these allowed crystallization assays in the 10–50 µl range, so that projects could be started with amounts of protein in the 1–100 mg range. However, the success rate of crystallization (*i.e.* the number of successful trials compared with the total number of trials) was poor, and in practice increasing numbers of trials and much larger amounts of proteins were required. Furthermore, for most crystallized proteins the time between the first crystal and resolution of the three-dimensional structure was long, and could reach several years. As a result, only a few structures had been solved by 1980, corresponding to <0.1% of the presently deposited structures in the Protein Data Bank (PDB).

### 2.2. Better crystallization methods and still a bottleneck   

For practical reasons and also as the outcome of interdisciplinarity, crystallization methods and strategies better adapted to proteins were invented and old forgotten methods were rejuvenated, for example crystallization in gelled media. This allowed the crystallization of ever-smaller amounts of protein *via* assays in ever-smaller volumes (from the microlitre to the nanolitre range). A diversified toolbox is now at the disposal of structural biologists (Table 1 ▸), enabling large-scale screening of crystallization parameters and growth optimization for enhancing the likelihood of obtaining well diffracting crystals (Giegé, 2013 ▸; Russo Krauss *et al.*, 2013 ▸; Sauter *et al.*, 2012 ▸). Note that the conventional methods (vapour diffusion, microbatch and incomplete factorial parameter screening) are still favoured by most experimentalists, and few structural biologists use more advanced methods. The latter, which are either physics-driven [for example counter-diffusion (Otálora *et al.*, 2009 ▸), gelled media (Lorber *et al.*, 2009 ▸), microfluidics (Maeki *et al.*, 2016 ▸) or stirring (Maki *et al.*, 2008 ▸)] or biology-driven [for example the crystallization of fusion proteins (Ting *et al.*, 2016 ▸) or novel co-crystallization strategies using chaperones selected from combinatorial libraries (Hipolito *et al.*, 2014 ▸; Pardon *et al.*, 2014 ▸)] are slowly infiltrating structural biology laboratories.

**Figure 2:**
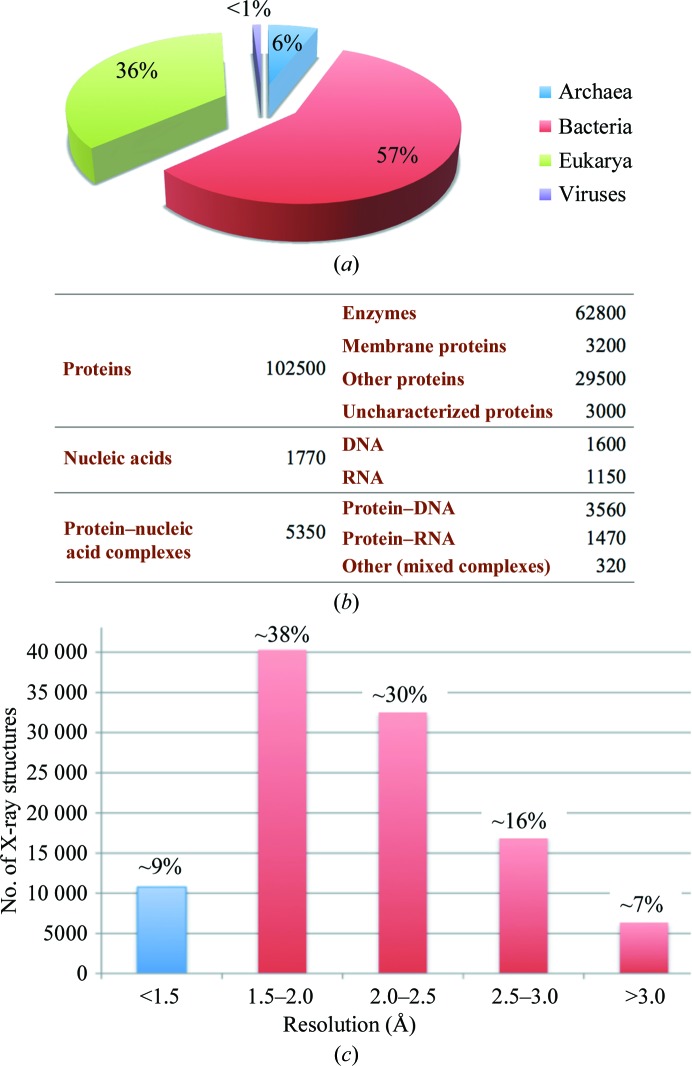
Qualitative and quantitative overview of X-ray structures. (*a*) Uneven coverage of the tree of life. (*b*) Incomplete coverage of macromolecular diversity. (*c*) Distribution of resolution. Among the known structures, 77% are solved at resolutions better than 2.5 Å, but only 9% are at resolutions better than 1.5 Å, notably ∼600 at subatomic resolution (≤1.0 Å); only two proteins (the small crambin and an iron–sulfur protein) have been solved at ultrahigh resolution (∼0.5 Å).

As a result, members of most protein classes and subclasses have been crystallized since the 1990s, and in the last two decades the number of successful crystallization attempts has increased tremendously, leading to the deposition of ∼1.1 × 10^5^ X-ray structures in the PDB. It could be falsely concluded that the crystallization bottleneck has been overcome. This is not the case, however, since these structures cover a restricted part of macromolecular diversity across the tree of life (Fig. 2 ▸ *a*). Although the structures of many protein families from the three kingdoms of life are represented, the coverage is uneven. Clearly, the structures of membrane proteins, nucleic acids and protein–nucleic acid complexes are under-represented (Fig. 2 ▸ *b*) and those of other proteins are over-represented (for example ∼550 structures of hen egg-white lysozyme and 250 of ferritins). Moreover, about half of the structures have been solved at medium or poor resolution, indicating that these structures were solved from crystals of rather weak diffraction quality (Fig. 2 ▸ *c*).

Why is there this partial coverage of macromolecular diversity? It is likely to be owing to the idiosyncratic attributes of biomaterials, such as inherent plasticity, hydrophobicity of membrane proteins, or physicochemical and architectural characteristics of RNAs. Moreover, many proteins include unfolded domains or are intrinsically unstructured (Dyson & Wright, 2005 ▸). To add to the difficulties, the attributes of protein families are not clear-cut, such as the ‘hydrophobicity’ partly present in soluble proteins, implying that the crystallization methods developed for membrane proteins can be applied to soluble proteins (Caffrey, 2015 ▸).

### 2.3. The quest to understand and optimize   

Since the early days of biological crystallography, pioneers have aimed to understand protein crystal growth. Today, it is accepted that the general principles governing the crystallization of inorganic materials also apply to the protein field (Vekilov & Chernov, 2002 ▸) and that diffusive mass transport during growth should be favoured, a condition that is unfortunately not fulfilled during crystallization by conventional methods. In addition, protein-specific parameters were found to be crucial, the most important being the protein itself (Giegé, 2013 ▸). Importantly, the two-step nucleation mechanism proposed for protein crystallization also applies outside the protein field, as critically discussed by experts in materials science (Erdemir *et al.*, 2009 ▸). Like in­organic crystals, protein crystals grow by screw dislocation at low supersaturation and by the formation of two-dimensional islands at higher supersaturation. However, uniform growth is not guaranteed in the currently used crystallization setups (*i.e.* in vapour diffusion), since supersaturation decreases during growth, so that a single crystal can grow by the two mechanisms, as explicitly seen with tRNA^Phe^ crystals (Ng *et al.*, 1997 ▸). This produces internal stress in the crystals and affects mosaicity, as has been shown with lysozyme (Chernov, 1999 ▸), and therefore adversely affects the practical use of many macromolecular crystals. A remedy could be crystal growth at constant supersaturation, as can be performed in flow cells, although this is unfortunately not user-friendly.

In the 1990s, when the demand for macromolecular crystals became crucial, pioneers tried to understand the factors that make many proteins resistant to crystallization. A few explored the inter-protein contacts in crystal lattices and compared them with the contacts occurring in oligomer interfaces. The examination of rather small sets of structures (∼200 PDB entries) showed subtle differences in size and amino-acid composition within contact patches, with a tendency for smaller patches rich in polar residues and looser interactions in packing contacts (Janin & Rodier, 1995 ▸). This suggested a harmful role for surface lysine residues in proteins that are resistant to crystallization (Dasgupta *et al.*, 1997 ▸).

Practical considerations motivated researchers to seek crystallization predictors and strategies to optimize the success rate of crystallization. Thus, physicochemical properties affecting crystal growth were sought from the crystallization data sets (≥500 proteins) collected by structural genomics consortia, for example for *Thermotoga maritima* (Canaves *et al.*, 2004 ▸) or from bacterial and human proteomes (Price *et al.*, 2009 ▸). This drew attention to well ordered surface patches that mediate inter-protein interactions and to less-ordered surface patches, with high surface entropy, that lower the crystallization propensity (Derewenda & Vekilov, 2006 ▸; Price *et al.*, 2009 ▸). In the same way, basic knowledge on crystal architectures was exploited to engineer proteins for enhanced crystallizability. Thus, knowledge of the packing contacts in ferritins guided the mutagenesis of human ferritin to enhance crystallization (Lawson *et al.*, 1991 ▸). Likewise, surface-entropy reduction strategies, in which surface lysine, glutamine or glutamate residues with mobile side chains are replaced by smaller alanine residues, produced crystallizable mutants of proteins (Goldschmidt *et al.*, 2014 ▸). In parallel, analyses of crystallization databases helped to optimize crystallization screens (Fazio *et al.*, 2014 ▸), for example using PEG-based cocktails (Chaikuad *et al.*, 2015 ▸). Another promising approach came from the improved success rate of liganded protein or nucleic acid crystallizations guided by biophysical diagnostics (Chung, 2007 ▸; Da Veiga *et al.*, 2016 ▸). Another advance came from knowledge of the physical chemistry of concentrated protein solutions, which revealed the practical importance of the second virial coefficient (*B*_22_). This coefficient informs about the attractive or repulsive interactions that favour or disfavour protein association under pre-crystallization conditions. Thus, slightly negative *B*_22_ values point to globally attractive interactions favouring crystallization and positive values indicate repulsive interactions favouring aggregation. Thereby, *B*_22_ became a useful predictor of the likelihood of proteins to crystallize (George *et al.*, 1997 ▸).

### 2.4. The present status of crystallogenesis   

Today, crystallogenesis is a discipline merging biology (mainly biochemistry and molecular biology), physics, chemistry and associated technologies, in which basic and applied aspects are equally important. Taking into account the chemical and structural peculiarities of biological macromolecules and given the universality of crystal-growth rules, methods have been devised to enhance the preparation of protein crystals suitable for structural biology. Yet, protein crystallization remains challenging, since most proteins are recalcitrant to crystallization.

Scattered reports over many years have suggested that the scope of macromolecular crystallogenesis extends beyond structural biology, but this perspective remains scarcely investigated. The existence of protein crystals that form *in vivo* and of proteic crystallites associated with human pathologies also pose questions regarding their formation in complex fluids that differ from the current crystallization media, in which protein purity is essential.

## 3. A pivotal period for breaking boundaries   

In the present pivotal time for biological sciences, the challenges of crystallogenesis require more interdisciplinarity. Thus, the classical physicochemical picture of protein crystal growth should be expanded to be more focused on chemistry and biology. This should lead to novel ways to grow crystals to solve structures that are missing from the PDB. Advanced strategies could help to enhance the crystallization of partly unstructured proteins (for example upon the binding of selected ligands) and to grow crystals of proteins in apo and transiently liganded forms. Understanding why and how protein crystals grow *in vivo* is another challenge. This aspect is highlighted by the recent interest in such crystals in structural biology (Boudes *et al.*, 2016 ▸). Finally, crystallization still needs to be refined for serial femtosecond and X-ray free-electron laser crystallography (SFX and XFEL; Levantino *et al.*, 2015 ▸) and for the renewed use of neutron crystallography (Blakeley *et al.*, 2015 ▸).

The time is also ripe to unify the outcomes of macromolecular crystallogenesis, which so far mainly concern structural biology, to integrate topics that are seemingly beyond the periphery of structural biology. Shifting the foundations of the discipline towards chemistry could do this. This implies a supramolecular vision of protein crystallization. If protein crystallization is seen as a self-assembly process, it can be approached from the conceptual background of supramolecular chemistry. As emphasized by Jean-Marie Lehn:

supramolecular chemistry aims at implementing highly complex chemical systems from molecular components held together by non-covalent intermolecular forces and effecting molecular recognition, catalysis and transport processes

(Lehn, 2012 ▸). This applies perfectly to macromolecular crystals, including crystals of viruses and huge molecular machines (note that catalysis can occur *in cristallo*). Molecular recognition, catalysis and transport processes are also keywords in molecular and cellular biology. Logically, this means that protein crystallization and molecular biology are based on similar supramolecular rules. Knowledge of such rules would immediately find application in understanding protein crystal growth in living organisms (Doye & Poon, 2006 ▸). Furthermore, it is tempting to suggest that protein crystals represent simplified model systems, from which can be deciphered general rules underlying the more complex molecular assemblies and functional processes occurring in living organisms. Going one step ahead, one can anticipate that macromolecular crystallogenesis will provide insights into an emerging supramolecular biology.

## 4. What is needed in the future?   

### 4.1. Prologue   

Two lines of research trace the future of macromolecular crystallogenesis. Firstly, efforts to improve the growth of crystals for structural biology in order to better understand the universe of proteins and RNAs, to refine the molecular-based taxonomy of species by filling gaps in the three-dimensional space of the tree of life and to understand four-dimensional structures (with time as the fourth parameter). Secondly, progress towards the world of supramolecularity with crystals seen as self-assembled entities with peculiar stability and plasticity.

Future developments will depend on technological and computational advances and will also benefit from structures solved by cryo-EM. For a rational choice of crystallization conditions, novel tools will facilitate comparison of the outcomes of crystallization experiments (Bruno *et al.*, 2014 ▸). Other tools are aimed towards a microscopic picture of the dynamics of protein crystals and a realistic modelling of crystallization conditions (Kuzmanic & Zagrovic, 2014 ▸) and, more radically, towards computational tools to guide crystallization (Altan *et al.*, 2016 ▸). Furthermore, new methods will help in the preparation of nanocrystals suitable for data collection on SFX and XFEL instruments (Boudes *et al.*, 2016 ▸). Also, the toolbox for mining structure and sequence databases is being enlarged, for example for automated evolution-based assessment of protein–protein interfaces (Baskaran *et al.*, 2014 ▸). Finally, the tools developed in soft-matter physics for studying colloidal assemblies will offer new insights into problems of protein crystallization (Fusco & Charbonneau, 2016 ▸).

### 4.2. Short-term perspectives within and outside structural biology   

#### 4.2.1. Towards a refined understanding of the structural landscape of proteins   

Different types of crystals are needed: firstly, crystals representative of protein families that are under-represented in or missing from the PDB and, secondly, crystals of proteins from taxonomic branches that are poorly represented or absent in the PDB, as well as crystals of the novel proteins that are continually being discovered from microbiotes and metagenomes. Also, crystals of proteins encoded in the genomes of giant viruses are expected. These will offer a timely opportunity to enter into the new biology of these viruses (Claverie & Abergel, 2016 ▸). The resulting structures will provide new insights into evolution and will allow a better correlation of genome-based or organism-based phylogenies with structure-based phylogenies.

Analysis of genomic databases, which are much larger than the PDB, should provide guidelines for pertinent choices of the proteins to be crystallized (for example from the proteomes of eukaryotic human pathogens). Also, the crystallization of prokaryal-like mitochondrial proteins, which are likely to be different from their human cytosolic homologues, is of interest for rational drug design and applications in medicine. On the other hand, since many protein structures are partly disordered, stable domains should be dissected from genes and overexpressed for crystallization. Flexible or unstructured proteins could also be stabilized in natural or artificial complexes that are more prone to crystallization. Using macrocyclic peptides (Hipolito *et al.*, 2014 ▸) or camelid antibody fragments (*i.e.* nanobodies; Pardon *et al.*, 2014 ▸) as specific co-crystallization chaperones selected from combinatorial libraries may be strategies of universal application.

Preparation of protein crystals of special biological interest in apo and liganded forms can be challenging. Such crystals with functional states that can be captured *in cristallo* are needed to uncover the dynamics of biological processes at the molecular level (for example during allosteric motions, conformational adaptations in complexes and enzymatic reactions). To develop plausible kinematic pathways based on sets of transient structures, crystal polymorphs are needed for each step in the functioning of the selected proteins. Resolution of their structures will allow functional effects (conserved in polymorphs) to be distinguished from packing ‘artefacts’ (not conserved). Finding the same effects in homologous proteins will provide additional support. Alternatively, time-resolved SFX and XFEL crystallography could bring quick answers, but is not of general application because only small motions can be detected in the crystalline environment and because few systems allow *in situ* initiation of their reactions concomitant with the X-ray pulses (Levantino *et al.*, 2015 ▸).

**Figure 3:**
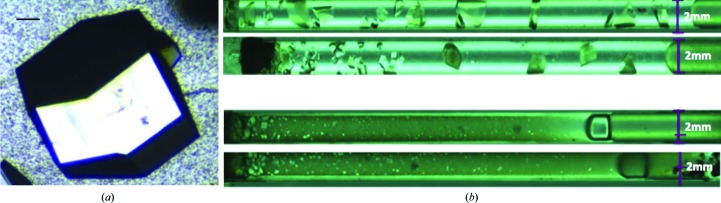
Images of large protein crystals for neutron crystallography grown by dialysis and counter-diffusion. (*a*) Crystal (volume of ∼1 mm^3^, scale bar 100 µm) of hen egg-white lysozyme grown in a temperature-controlled dialysis flow cell of 80 µl (courtesy of M. Budayova-Spano). (*b*) Crystals of inorganic pyrophosphatase from *Thermococcus thioreducens* grown in counter-diffusion capillaries of inner diameter 2 mm and length 50 mm (starting precipitant on the left; courtesy of J. Ng). The largest crystals (volume of ∼5 mm^3^) grew under microgravity. The gradient of supersaturation anticipated in counter-diffusion experiments is clearly seen in the capillaries of microgravity-grown crystals (top, with crystals of increasing size from the left to the right), but is absent in the capillaries of Earth-grown crystals (bottom) because of harmful convection in the large capillaries.

Another aspect concerns the optimization of crystal perfection. For this purpose, several approaches are possible, most based on crystal growth under diffusive regimes (for example counter-diffusion and gelled media). To guarantee growth under a uniform regime, control of the thermodynamics and kinetics at all stages of crystallization is needed. Diagnostic tools [for example dynamic light scattering (DLS), small-angle X-ray scattering (SAXS), calorimetry, interferometries and microscopies] and computational database mining are essential to uncover the relative importance of the crystallization parameters. Finally, efficient methods to generate tiny crystals for SFX and XFEL crystallography and large crystals for neutron crystallography are needed. Protein nanocrystals can be found and characterized in reversible precipitates or produced on purpose, for example in special batch systems controlled by DLS (Schubert *et al.*, 2015 ▸). Large crystals can be grown by methods based on dialysis or counter-diffusion (Fig. 3 ▸). In one strategy, crystallization occurs in a temperature-controlled flow-cell dialysis system (Junius *et al.*, 2016 ▸). In another, large crystals are grown by counter-diffusion and Ostwald-like ripening in capillaries of large diameter. Because of their large diameter, diffusive mass transport is not optimal on Earth, but can be enhanced under so-called microgravity environments, thereby leading to enhanced crystal size and perfection (Ng *et al.*, 2015 ▸).

#### 4.2.2. Implications for materials science, biotechnology and molecular medicine   

A few applications of these ideas outside structural biology have already emerged and others are awaited. For instance, antibodies could be used as sensors of two-dimensional and three-dimensional organized crystalline surfaces. Recognition of crystals composed of relatively small organic molecules, such as cholesterol monohydrate, by selected monoclonal antibodies provided a proof of concept (Addadi *et al.*, 2008 ▸). The concept may apply to protein crystals, but this awaits confirmation.

Self-assembly rules will guide the engineering of novel nanoscaled materials. For nucleic acids, the idea was proposed by Seeman in the 1980s and has been exploited for the preparation of self-assembled DNA crystals (Zheng *et al.*, 2009 ▸) and of photonic crystals developed through DNA-programmable assembly (Park *et al.*, 2015 ▸). Such crystals can have multiple applications in biology and materials science (Jones *et al.*, 2015 ▸). In the protein field, self-assembly processes are ubiquitous, but have attracted attention only recently. Seen from a soft-matter perspective, their study is essential to understand protein-condensation diseases (*e.g.* Alzheimer’s disease) and biotechnological purification processes based on liquid–liquid phase separation, as well as being important for protein crystallization (McManus *et al.*, 2016 ▸).

Engineering protein crystals is another approach. One method aims to deliver crystals of fusion proteins constituted of a cargo (the Cry3Aa protein from *Bacillus thuringiensis*) fused to reporter proteins. Such fusions grown in the bacterium can be taken up *in vitro* by different cell lines or delivered to mice *in vivo* *via* many modes of administration (Nair *et al.*, 2015 ▸). In another strategy, protein crystals are functionalized *in vitro* with metallic or organic compounds, as shown with ferritin and lysozyme crystals that were converted into catalytic, magnetic, luminescent or fluorescent nanoparticles (Abe *et al.*, 2016 ▸).

### 4.3. Trends towards supramolecular crystallogenesis   

#### 4.3.1. Supramolecularity guided by chemistry and physical chemistry   

Self-assembly and supramolecular processes are seminal attributes of life. Therefore, an integrated understanding of supramolecularity implies that macromolecular entities that crystallize or participate in biochemical processes require discrete physical and chemical determinants and antideterminants that favour or disfavour correct or incorrect molecular recognition. The search for determinants appears to be most immediate, and is well documented, in biochemistry (*e.g.* Giegé & Eriani, 2014 ▸), but is much less so in crystallogenesis. Antideterminants, on the other hand, are poorly known, although the first findings came from studies performed in the 1990s on the crystallization of ferritins (Lawson *et al.*, 1991 ▸). Thus, analysis of the packing contacts in horse ferritin crystals and the sequence comparison of three ferritins indicated that Asp84 and Gln86 are crystallization determinants and that Lys86 is an antideterminant. Given the conceptual similarities to biochemistry, this suggests that the recognition patterns in proteins or nucleic acids interacting in crystal lattices and in solution are in part similar.

Approaching the question of molecular recognition from the perspective of physics should provide global answers. However, until now the noncovalent binding thermodynamics of inter-protein interactions have essentially been addressed separately for proteins in the crystalline state and in solution. Consequently, an integrative understanding that merges both crystal and solution aspects has not yet emerged. Nevertheless, it has been proposed that proteins in the crystalline state are more stable than in solution (Drenth & Haas, 1992 ▸) and that low side-chain entropy of surface residues is a significant determinant of crystallization propensity, although this propensity is not strongly influenced by the overall thermodynamic stability of proteins (Price *et al.*, 2009 ▸). Recently, based on soft-matter physics, it has been shown that the geometrical asymmetry of patches on protein surfaces weakly affects crystallization, in contrast to the bond-energy asymmetry that markedly interferes with crystallization thermodynamics and kinetics (Fusco & Charbonneau, 2013 ▸).

A promising connection between solution and crystal physics came from a mechanism for controlled protein interactions mediated by multivalent metal cations that activate attractive patches on protein surfaces, thereby facilitating the formation of inter-protein ion bridges in solution and in crystals (Roosen-Runge *et al.*, 2014 ▸). The implication is that inter-protein ion bridges favour crystallization. A crystallization method termed ‘metal-mediated synthetic symmetrization’ supports this possibility, since the introduction of histidine or cysteine residues on protein surfaces for coordination with metal ions triggers crystallization (Laganowsky *et al.*, 2011 ▸). This is reminiscent of the old observation on the role of Ca^2+^ ions that mediate the crystallization of ferritins (Lawson *et al.*, 1991 ▸).

Other openings have come from data mining of the PDB. Comparison of packing interfaces and biological interfaces in monomeric and homodimeric proteins showed that large crystal-packing contacts have interface areas and contact sizes similar to those of permanent homodimers (Table 2 ▸). The properties of these packing contacts show similarities to the weak transient complexes occurring in nonpermanent homodimers, as reflected by the number of hydrogen bonds and noncovalent contacts (ionic, hydrophobic, π and van der Waals). Moreover, packing contacts appear to be more loosely organized, with less hydrophobic interactions than in the permanent subunit contacts in homodimers (Luo *et al.*, 2015 ▸). Generalizing these conclusions is presently premature since the analyses were performed on a limited number of structures. However, the trend is promising and calls for further computational studies to better comprehend the structural principles underlying packing or recognition rules. For this purpose, the amino-acid or nucleotide residues involved in crystal formation owing to direct or indirect (metal-ion or water-mediated) weak noncovalent interactions should be more systematically characterized on larger and well defined macromolecule families.

On the experimental side, the engineering of protein surfaces or the mutagenesis of packing contacts in crystals is needed to decode the recognition rules underlying protein crystallizability. Several proof-of-concept studies provide guidelines for the future. Thus, a preliminary study in the 1990s on the crystallizability of thymidylate synthase showed that mutating single surface amino acids yields crystal polymorphs with dramatically altered solubilities (McElroy *et al.*, 1992 ▸). Another study showed that the bovine pancreatic trypsin inhibitor crystallizes in polymorphs assembled from monomers or decamers as the result of oligomeric changes that occur under pre-crystallization conditions (Hamiaux *et al.*, 2000 ▸). Also noteworthy was the engineering of the ParB-like nuclease by reductive methylation of surface lysine residues, which created new intermolecular and intramolecular contacts and thus resulted in well diffracting crystals with enhanced packing and protein stability (Shaw *et al.*, 2007 ▸).

Neglected but essential aspects concern the solvent content of macromolecular crystals and solvent effects that influence the activity, stability and intermolecular interactions of macromolecules. Solvent constitutes about half of the crystal volume on average, and up to 80% in extreme cases. Dis­ordered bulk solvent allows *in cristallo* molecular flexibility and in some cases even functional activity. However, part of the solvent is ordered and forms a hydration shell around proteins (Kim *et al.*, 2016 ▸; Weichenberger & Rupp, 2014 ▸). This shell has a patchy organization, which is likely to be complementary to the patches on protein surfaces. According to SAXS and SANS data, solvent density modifications occur at protein surfaces, as seen for the green fluorescent protein. Thus, the hydration shell is locally denser in the vicinity of acidic surface residues, while hydrophilic, hydrophobic and basic residues modify the density only mildly. These modifications result from the combined effects of residue-specific ion recruitments from the bulk solution and water structural rearrangements (Kim *et al.*, 2016 ▸). Similarly, in crystals containing nucleic acids, both anions (D’Ascenzo & Auffinger, 2016 ▸) and cations (Auffinger *et al.*, 2016 ▸) stabilize the structure of DNA or RNA.

#### 4.3.2. Supramolecularity guided by evolution and integrative biology   

Several facts directly related to evolution have to be taken into account to understand macromolecular crystallization. Firstly, evolution has only explored a small part of the potential diversity of the sequence space of proteins and nucleic acids. Ancient sequences were likely to be short and compatible with folding into more or less stable conformers. In the course of evolution these sequences became larger, mainly by the fusion of structural domains. This is well exemplified when comparing proteins with the same function from bacteria and higher eukarya. Secondly, protein crystals have been observed in many organisms from all kingdoms of life, indicating that crowded biological fluids are not inevitably harmful for protein crystallization (Doye & Poon, 2006 ▸). This fact, which seemingly contradicts the current belief that purity favours crystallization, was long neglected. Nevertheless, most proteins remain soluble *in vivo*. This contrasting truth has been conceptualized in the ‘evolutionary negative design’ principle (Doye *et al.*, 2004 ▸). Thus, protein sequences and physicochemical parameters are proposed to have evolved to avoid crystallization in biological media. If the molecular composition and physicochemical parameters of these media are modified, crystallization or aggregation of proteins may occur. This implies a balance between features that favour or disfavour self-association for each protein present in a given biological fluid. If this balance is broken by modification of protein concentration, mutation or a change in the physicochemical properties of the fluids, functional disorders may occur (for example in human diseases associated with crystalline or aggregated phases). Such functional disorders often occur in expression systems in which inclusion bodies can contain aggregated or microcrystalline proteins, especially when the expression levels of the target proteins are too high.

Furthermore, and as a consequence of sequence properties, evolution has determined the architecture, structural stability and dynamics of proteins, as well as the structural organization of multi-macromolecular systems. Symmetry and especially asymmetry are often invoked when describing such systems. Thus, structural symmetry provides stability, as in spherical viruses or in ferritins, but is rare, while asymmetry is frequent and occurs in dynamic systems (Blundell *et al.*, 2002 ▸). In other words, as quoted by art historians, ‘symmetry signifies rest and binding and asymmetry motion and loosening’ (McManus, 2005 ▸). These attributes apply to crystallogenesis, since the crystallization of symmetric structures is easier than that of asymmetric structures, as reflected by the over-representation of symmetric structures in the PDB. Consequently, symmetry should stabilize crystals and help crystallization. This conjecture was verified by crystallizing proteins after symmetrization (Laganowsky *et al.*, 2011 ▸). Likewise, deliberate reduction of asymmetry should help crystallization, for example by the removal of post-translational modifications. This was first verified in the 1990s by the crystallization of glycoproteins after enzymatic deglycosylation (Baker *et al.*, 1994 ▸). However, this is at the expense of lost biological information, and hence novel methods to overcome this bottleneck are awaited. This is especially true for post-transcriptionally modified RNAs.

When supramolecular crystallogenesis is seen from the viewpoint of integrative biology, one has to consider the consequences of cellular crowding on protein self-assembly. This is challenging, since the total concentration of protein and RNA inside, for example, an *Escherichia coli* cell is ∼300–400 mg ml^−1^ (Ellis, 2001 ▸). Despite such conditions, individual proteins and even large macromolecular assemblies can crystallize in crowded media (Doye & Poon, 2006 ▸). On the other hand, proteins participate in many inter-protein associations in crowded media, as is found in interactomes. For instance, many binary protein–protein interactions have been identified for the 726 proteins encoded in the small genome of the syphilis spirochete *Trypanema pallidom* (Titz *et al.*, 2008 ▸). Yet, precise characterization of the contact patches that mediate these interactions is difficult, since only 36 crystal structures from the spirochete are present in the PDB. Other interactomes list partners of important target proteins, such as the phosphatase from *Plasmodium falciparum*, an enzyme that is essential for the viability of the parasite. This enzyme interacts with 134 partners (Hollin *et al.*, 2016 ▸), but among the 530 known three-dimensional structures of plasmodial proteins that of the phosphatase is missing as well as those of most of its interaction partners. These examples show that many more three-dimensional structures are needed to assess the structural basis of the protein–protein interactions in interactomes.

Altogether, this points to the significance of the patchy organization of protein surfaces. It is likely that surface patches represent evolutionary adaptations to sustain the multiple interactions that proteins make during cellular life, either for structural or functional reasons. This leads to a universe of interactions, which are essentially uncharacterized, and gives sense to the variable strength of inter-protein or inter-patch contacts. Obviously, the interplay of these interactions is crucial for crystallization (Fusco & Charbonneau, 2013 ▸).

These considerations emphasize the need to understand the organization of matter in living systems. Clearly, the location of proteins is not completely disordered in biological fluids and their ordering is necessarily enhanced in crystals. In between, proteins can occur in a variety of mesophases (lyotropic membranes, two-dimensional crystals, liquid crystals, spherulites and other paracrystallites). In such intermediate states between solid and liquid, the principal molecular properties of ordered structures are maintained, but structural rigidity decreases while kinetic disorder and entropy increase. Such possibilities were suspected long ago (Bernal & Crowfoot, 1933 ▸), but only recently has their biological importance been appreciated (Hyde, 2015 ▸) with attempts to gain knowledge from a soft condensed matter perspective (McManus *et al.*, 2016 ▸). At present, most questions about the fate and organization of matter under biologically relevant conditions await answers. For example: what are the proteins that are recalcitrant to crystallization *in cellulo*? Or can all proteins crystallize *in vivo*? To what extent are contact patches involved in crystal packing also involved in interactome interactions? What are the proteins most commonly found in macromolecular assemblies? What are the transient assemblies mostly found *in vivo*?

Given the diversity of possible interactions, it is worthwhile identifying the supramolecular parameters that modulate the strength of the inter-patch contacts and deciphering their exact chemical nature. This requires precise computational analyses of protein surfaces and more crystals (with interactome-guided selection of the proteins) for structural analyses.

### 4.4. Epilogue: towards supramolecular biology and generalized crystallogenesis   

To conclude, a visionary sentence, written 25 years ago in a paper on the stability of protein crystals, must be highlighted:

Protein crystals are not only important for the crystallographer, but they have more virtue. One day they may even play a role in the material sciences or in electric circuits…

(Drenth & Haas, 1992 ▸). Today, this prophecy has been fulfilled and new disciplines percolate macromolecular crystallo­genesis, such as supramolecular chemistry (Uhlenheuer *et al.*, 2010 ▸) and soft-matter sciences (Fusco & Charbonneau, 2016 ▸). In a wider perspective, self-assembly recognition rules open the route to supramolecular biology. Here, one is faced with a continuum of interactions, from strong to weak (essentially transient), occurring in cellular mesophases. Phase transitions play crucial roles in such self-assembly processes (Lee *et al.*, 2013 ▸). Understanding packing and oligomer contacts should provide operational models for a wider understanding of functional *in cellulo* interactions. To this end, physical and chemical determinants and antideterminants that control self-assembly will be crucial.

Finally, the intricate organization of biomolecular entities in dynamic mesophases points to the notion of structural order in biological systems. Such order correlates with the existence of different types of symmetric and pseudo-symmetric patterns that are regularly found in biosystems ranging from microscale and nanoscale patterns (within proteins and nucleic acids, oligomers, viruses and multi-macromolecular assemblies) up to macroscale patterns (within cellular and organismic phenotypes). The need to understand their nature and genesis explains the emergence of a generalized crystallography (Hyde, 2015 ▸) and, better, of a generalized crystallogenesis where biology, chemistry, physics and even aesthetics are intimately interwoven. 

## Figures and Tables

**Table:** (*a*) Crystallization methods.

Method	Comments	Proof of concept (year)
Early micro-methods		
Dialysis	>1 ml down to 4 µl	1959
Vapour diffusion	2–50 µl	1968
Batch	Millilitres down to <2 µl	1971
Interface diffusion	Diameter of microtubes <6 mm	1972
A few advanced methods		
Gelled media	Favours mass transport by diffusion (operates in all devices)	1954
Growth on surfaces	Induction of nucleation on modified surfaces	1992
Counter-diffusion	Favours mass transport by diffusion (operates in capillaries)	1993
Microfluidics	Favours mass transport by diffusion	2002
Stirring	Improves resolution and mosaicity	2002
Laser light pulses	Cavitation induces nucleation	2003
Gel and laser pulses	Enhances nucleation	2013
Adsorption and desorption	Improves success rate and crystal quality	2014

**Table:** (*b*) Crystallization strategies.

Strategy	First application (year)
Early strategies	
Limited proteolysis	1971
Homologous proteins from thermophiles	1973
A few advanced strategies	
Detergents for membrane-protein crystallization	1980
Seeding	1981
Co-crystallization with antibodies	1983
Automation	1990
Sparse-matrix sampling	1991
Protein engineering	1991
Temperature as a variable	1992
Mutagenesis for surface-entropy reduction	2001
Nanobodies as crystallization chaperones	2009
Macrocyclic peptides as co-crystallization ligands	2013

**Table 2:** Towards understanding the geometric and physicochemical properties of the protein surfaces seen in crystals: a precursory proof-of-concept study with a view to finding crystallization indicators Adapted from Luo *et al.* (2015 ▸). Asterisks indicate statistical significance.

	Protein–protein packing interactions (in 773 monomeric structures)		‘Specific’ protein–protein interactions (in 117 homodimeric structures)†	
Interface features	General crystal-packing contacts	Large crystal-packing contacts	Weak transient complexes	Permanent homodimers
Interface area (Å^2^)	531*	1472	718*	1950*
Interface area ratio (%)	56	61	9	16
No. of interface residues	35	84	42	113
No. of interface atoms	115	306	151	400
No. of nonbonded contacts	52*	169	83*	216*
No. of hydrogen bonds	2	7	4	10
No. of protein crystal structures in PDB	681	92	103	113

† The meaning of ‘specific’ refers to protein–protein interactions that differ from packing interactions (this meaning will evolve in the future when protein surfaces are better understood).

## Footnotes

1 Note: the term ‘protein’ is often taken as the generic name for a biological macromolecule or macromolecular assembly.
